# The implication of tumor biomarker CA19-9 in the diagnosis of intracranial epidermoid cyst

**DOI:** 10.18632/oncotarget.12934

**Published:** 2016-10-26

**Authors:** Yongjie Wang, Wei Yan, Qun Wu, Gao Chen, Jianmin Zhang

**Affiliations:** ^1^ Department of Neurosurgery, 2nd Affiliated Hospital, School of Medicine, Zhejiang University, Hangzhou, Zhejiang, China; ^2^ Department of Neurosurgery Brain Research Institute, Zhejiang University, Hangzhou, Zhejiang, China; ^3^ Department of hospital, school of medicine, Collaborative Innovation Center for Brain Science, Zhejiang University, Hangzhou, Zhejiang, China

**Keywords:** epidermoid cyst, CA19-9, ROC curves, diagnosis

## Abstract

**Object:**

The diagnosis of intracranial epidermoid cyst (IEC) relies solely on MRI, which is time and money consuming. The application of tumor biomarkers in IEC has never been systematically studied. Here we screened a group of commonly used tumor biomarkers to assess their diagnostic value in IEC.

**Results:**

Serum tumor biomarkers were assessed in 42 IECs and 42 paired healthy controls. Only serum CA19-9 level was significantly higher in the IEC group (median 20.3U/ml vs. 6.5U/ml, *p* < 0.001). Area under curve for CA19-9 was 0.806 (95% CI 0.700–0.912), with cutoff value of 13.15 U/ml (sensitivity 71.4%, specificity 97.6%). Tumor size was significantly different between CA19-9 positive and CA19-9 negative groups(64.14 ± 67.91cm3 vs. 19.43 ± 13.76 cm^3^, *p* = 0.04) and linear regression analysis revealed a positive correlation. Neither the extent of resection nor recurrence rate showed any significant difference between the two groups.

**Methods:**

This is a retrospective study of IEC patients treated between 2009 and 2014. We analyzed the expression of common serum tumor biomarkers, including carbohydrate antigen 19–9 (CA19-9), carcinoembryonic antigen, carbohydrate antigen125 and squamous cell carcinoma in both IEC and healthy control group. Receiver operating characterisitics curves were constructed to evaluate the diagnostic accuracy.

**Conclusions:**

Our data indicated that for serum CA19-9 level higher than 13.15U/ml, after excluding the possibility of gastrointestinal system tumor, lung cancer, inflammation and other related diseases, the existence of IEC should be considered. Further prospective study is needed to gain more understanding of the value of CA19- 9 in postoperative evaluation and surveillance.

## INTRODUCTION

Intracranial Epidermoid cyst (IEC), also known as cholesteatoma, is a rare type of primary intracranial benign tumor, originating from entrapped ectodermal tissue during embryogenesis [1]. IEC is most commonly found in the cerebellopontine angle and adheres closely to the surrounding neurovascular structures [2].Radiologically, IEC presented as an irregular non-enhanced mass spreading along the cisterns and characteristically displayed restricted signal on diffusion weighted image (DWI) [2, 3]. The diagnosis and post-operational follow-up of IEC relies solely on MRI, which is time and money consuming. Sometimes it is still difficult to differentiate IEC from other cystic lesions.

Serum tumor biomarkers have been applied to assist the diagnosis, prognostic evaluation and follow-up of certain types of tumors, which is cheap, convenient and efficient compared to radiologic evaluation. While for intracranial primary neoplasm, only β human chorionic gonadotropin (HCG) and α feto-protein (AFP) are proved to effectively indicate germ cell tumor [4]. Carbohydrate antigen 19-9 (CA19-9) is a type of glycoproteins located in epithelium of pancreatic and bile ducts, and has been found in the folliculo-tubular structures of normal and neoplastic pituitary [5]. In a small group of 7 patients diagnosed with IEC or dermoid cyst, Takeshita M. found that the preoperative serum levels were mildly to moderately elevated [6]. Others have reported abnormally high serum CA19-9 level in splenic ECs [7–9]. All these trivial evidences point to the possible diagnostic value of CA19-9 in IEC. Carcinoembryonic antigen(CEA) is primarily used to aid in the diagnosis of colon cancer and lung cancer. Squamous cell carcinoma antigen (SCC) belongs to glycoprotein composed and excreted by squamous cell carcinoma. Carbohydrate antigen 125 (CA125) is usually applied in evaluating ovarian tumors [10]. These tumor biomarkers share the common characteristics of originating from epithelium tissue.

To investigate this further, we compared the expression of several common serum tumor biomarkers including CA19-9 between IECs and normal controls. The diagnostic sensitivity, specificity and clinical significance of the positive candidates were further examined.

## RESULTS

**Figure 1:**
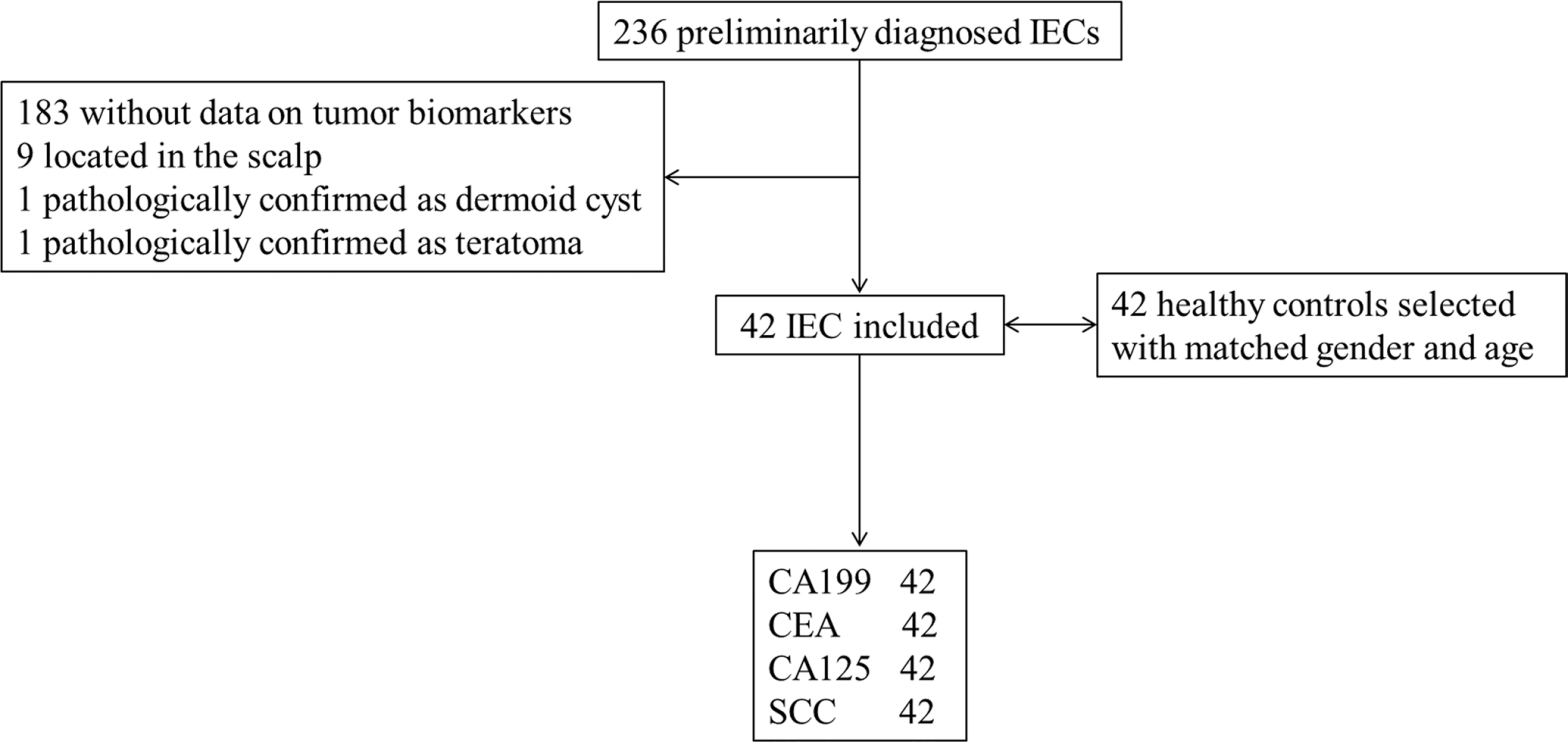
Study profile

There were 236 patients who were diagnosed with EC between 2009 and 2014. Excluding 183 cases without tumor biomarkers recorded, 9 cases located subcutaneously, 1 case of dermoid cyst and 1 case of teratoma, there were 42 patients finally enrolled. 42 healthy controls were matched by age and gender by an author blinded to the tumor biomarker results (Figure 1).

**Figure 2:**
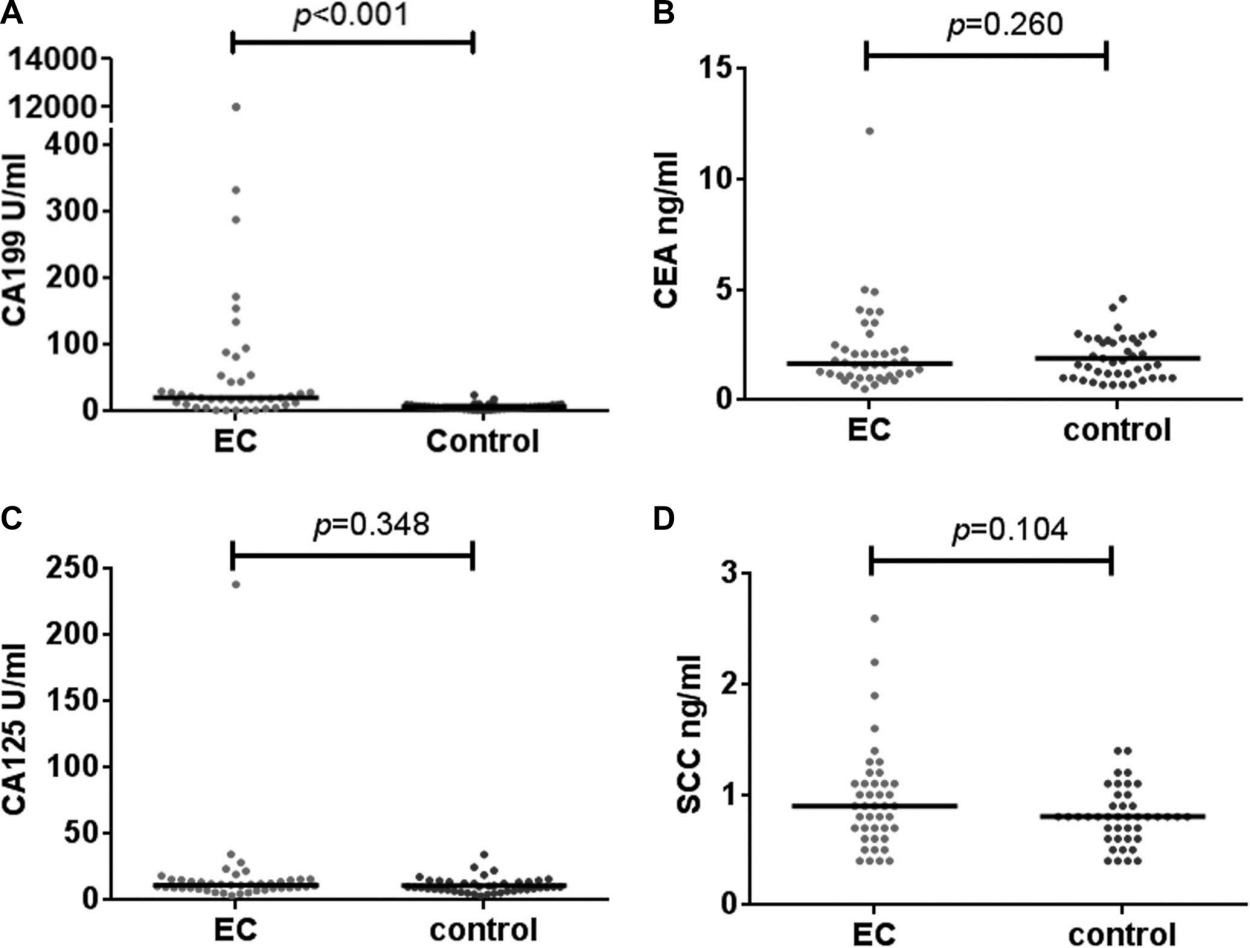
Differential median serum concentrations of tumor biomarkers between IEC and healthy controls The serum level of CA19-9 in IEC was significantly higher than in the healthy control group (**A**). While for CEA (**B**), CA125 (**C**) and SCC (**D**), the difference was insignificant.

**Figure 3:**
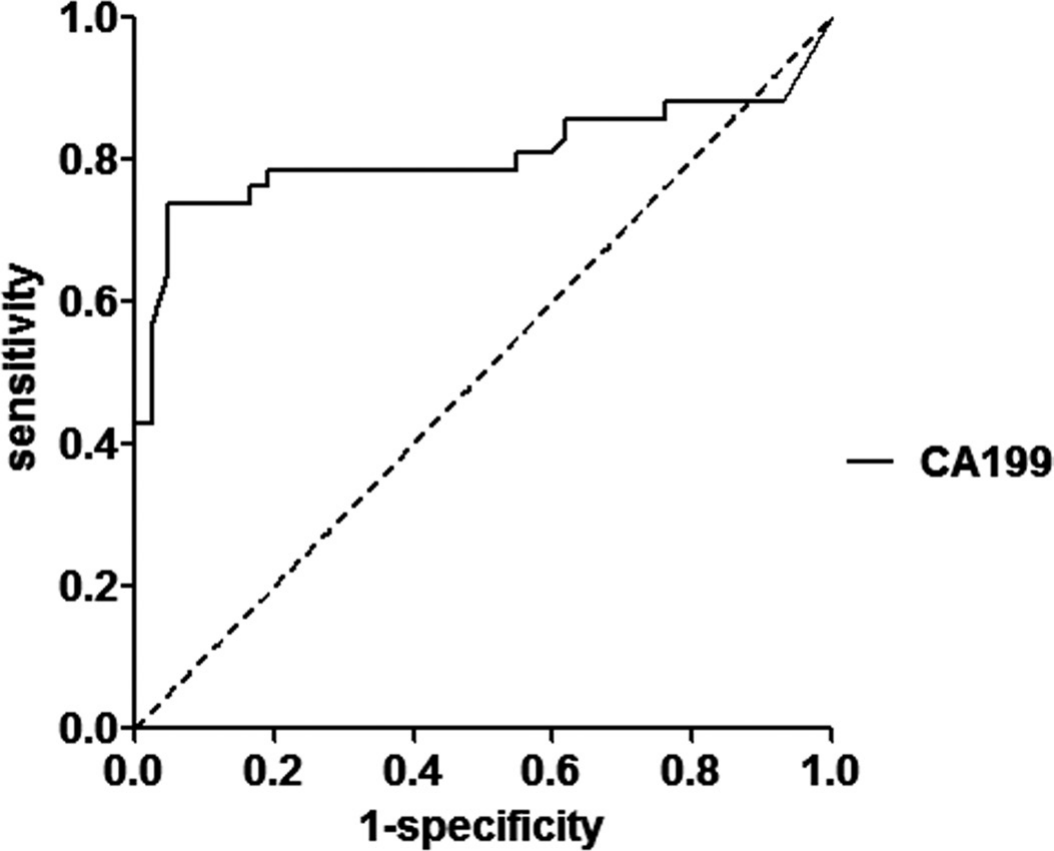
ROC curve for the serum CA19-9 in the diagnosis of IEC

Four epithelium originated tumor biomarkers, CA19-9, CA 125, CEA and SCC were compared across the two groups, CA19-9 were significantly higher in patients with IEC (mean 339.7,SD 2082.3 U/mL; median 20.3 ng/mL, IQR 39.1)than in healthy controls (mean 7.0,SD 4.1 U/mL; median 6.5 ng/mL, IQR 5.0)(*p* < 0.001; Table 1, Figure 2). ROC curves showed the optimum diagnostic cutoff for CA19-9 was 13.150 U/ml (AUC 0.806, 95% CI 0.700-0.912, sensitivity 71.4%, specificity 97.6%; Figure 3). Predictive values and likelihood ratios are shown in Table 2.

**Table 1:** Comparison of serum tumor biomarker expression between intracranial epidermoid cyst and healthy controls

	IEC		Healthy controls		Mann-Whitney U
	mean ± SD	median(IQR)	mean ± SD	median(IQR)	*p*
age/year	41.4 ± 15.1	43(21.0)	40.9 ± 14.6	43.0(20.0)	0.392
gender (male/female)	17/25		17/25		
Tumor biomarker					
CA19-9 U/ml	339.7 ± 2082.3	20.3(39.1)	7.0 ± 4.1	6.5(5.0)	< 0.001
CEA ng/ml	2.2 ± 2.0	1.6(1.2)	1.9 ± 1.0	1.7(1.8)	0.96
CA125 U/ml	18.0 ± 6.8	11.1(6.4)	11.7 ± 6.0	9.6(6.4)	0.138
SCC ng/ml	1.0 ± 0.5	0.9(0.5)	0.8 ± 0.3	0.8(0.3)	0.096

IEC: intracranial epidermoid cyst; SD: standard deviation; IQR: interquartile range; CA19-9: carbohydrate antigen 19-9; CEA: carcinoembryonic antigen; CA125: carbohydrate antigen125; SCC: squamous cell carcinoma

**Table 2:** Results for the measurement of serum CA19-9 in the diagnosis of intracranial epidermoid cyst

Tumor biomarker	AUC (95% CI)	Cutoff value	sensitivity %	specificity %	PPV	NPV	PLR	NLR
CA19-9	0.806 (0.700–0.912)	13.150	71.4%	97.6%	97.0%	77.0%	30.00	0.29

CA19-9: carbohydrate antigen 19-9; AUC: area under curve; CI: confidence interval; PPV: positive predictive value; NPV: negative predictive value; PLR: positive likelihood ratio; NLR: negative likelihood ratio.

**Figure 4:**
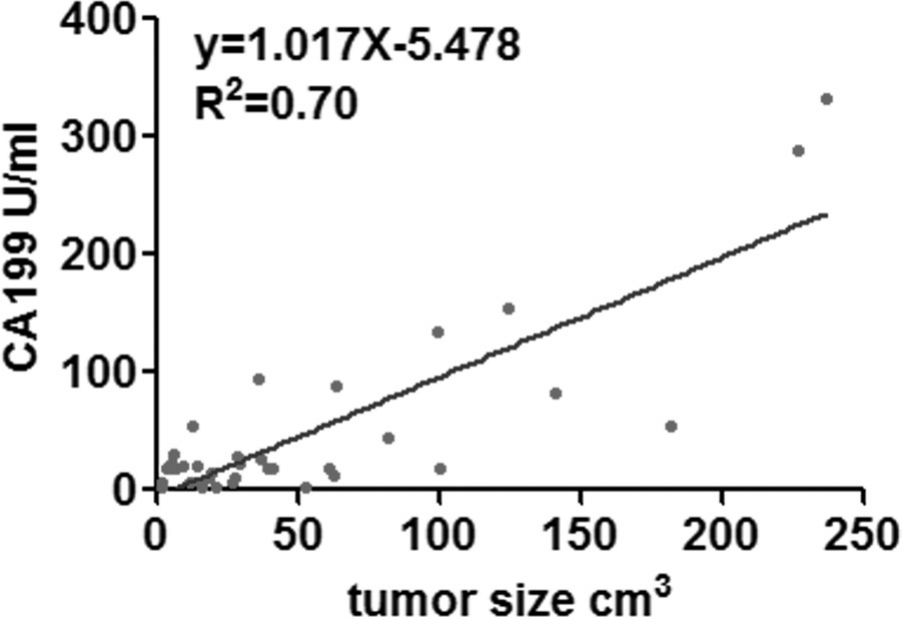
Linear regression analysis indicating a positive correlation between CA19-9 and tumor size

According to the above cutoff value, there were 31patients in the CA19-9 positive group, 11 patients in the CA19-9 negative group. One patient was excluded from the analysis for factors influencing CA19-9 expression, since had multiple lesions spreading along ventricular system, thus rendering the determination of size and location impossible. Both univariant and logistic regression analyses showed that the serum concentration of CA19-9 was significantly correlated with tumor size (*p* = 0.044, OR = 1.045, 95% CI 1.001–1.090; Table 3). Linear regression analysis revealed a positive correlation(R^2^ = 0.70; Figure 4). The serum concentrations of CA19-9 did not differ significantly with age (*p* = 0.845), gender (*p* = 0.241), first onset or recurrence (*p* = 0.733), or tumor location (*p* = 0.301) (Table 3).

**Table 3:** Univariant and logistic regression analysis for factors related to the expression of CA19-9

Factors	CA19-9 level			Logistic regression(Forward LR)		χ^2^	rank
	positive	negative	*p*	OR	95% CI	*p*	*p*
age/year (mean ± SD)	42.0 ± 14.8	39.0 ± 16.8	0.845				0.828
gender (male/female)	9/21	7/4	0.241			0.052	
size/cm^3^ (mean ± SD)	64.14 ± 67.91	19.43 ± 13.76	0.044	1.045	1.001–1.090		0.04
new/recurrence	27/3	8/3	0.733			0.187	
Location			0.301				0.638
Cerebellopontine angle	18	8					
parasellar	4	0					
parenchymal	5	1					
spine	1	1					
mastoid process	2	1					

CA 19-9:carbohydrate antigen 19-9; LR: logistic regression; OR: odds ratio; CI: confidence interval; SD: standard deviation;

Gross total resection was achieved in 12 patients (38.75%) among CA19-9 positive group, while 4 (36.4%) among CA19-9 negative group. The extent of resection did not show significant difference between the two groups. A total of 24 patients returned to the clinics during the mean period of 7.47 months. Recurrence occurred in 5 patients (27.8%) in the CA19-9 positive group, compared with 0 in the CA19-9 negative group. But the difference was insignificant (*p* = 0.07)(Table 4).

**Table 4:** The extend of surgery resection and recurrence during follow-up between CA19-9 positive and negative groups

			CA19-9	
		positive	negative	*p*
surgery	GTR	12	4	1.0
	STR	19	7	
Follow-up	recurred	5	0	0.07
	stable	13	6	
	durance/month (mean ± SD)	8.77 ± 11.11	4.67 ± 6.15	0.101

CA19-9: carbohydrate antigen 19-9; SD: standard deviation.

## DISCUSSION

Many tumor biomarkers have been established as useful tests in cancer diagnosis, treatment assessment and follow up surveillance [11]. One such biomarker, CA19-9, belonging to glycoproteins, when elevated beyond its clinical upper limit of 37.0 U/ml, has selective implication of gastrointestinal cancers, particularly of pancreatic cancer [12]. In this retrospective study, we have shown that measurement of serum CA19-9 has diagnostic value for IEC, with cutoff value of 13.15 U/ml. Tumor size was positively correlated with the expression of CA19-9. An insignificant trend of increased recurrence rate was found in the CA19-9 positive group.

EC can grow anywhere of the body, including palm, oral cavity, middle ear, neck, kidney, gastrointestinal tract, testis, etc., and about 7% are located in the head and neck [5, 6]. IEC is rare, accounting for only 0.2–1.8% of all intracranial neoplasm, and commonly affects people between 15 and 50 years old. Half of the lesions are located in the cerebellopontine angle. Occurrence in the parasellar region, basal cistern, sylvian fissure, pineal region and ventricle systems has also been reported [2, 13]. It has been hypothesized that IEC originates from the ectodermal tissue implanted during neural tube closure, or brought in by lumbar puncture, traumatic brain or spine injury [1, 14]. Histopathologically, EC is lined by simple or stratified squamous epithelium, covered by an outer layer of collagenous connective tissue. Cystic contents include debris, keratin and cholesterol without any skin appendages [2]. Considering the epithelial characteristic of EC, we only focused on four commonly applied tumor biomarkers, CA19-9, CA125, SCC and CEA, which are originated and secreted by epithelium tissue.

Abnormally high CA19-9 was reported in splenic ECs [7–9]. There has also been evidence of positive CA19-9 staining in craniopharyngioma, rathke cyst and cystic pituitary adenoma [5]. It has been firstly described by Takeshita in a group of 4 IEC patients that preoperative CA19-9 was mildly to moderately elevated in 2, and significantly dropped after surgical resection [6]. But as far as we know, concomitant diseases such as cancers of digestive system, inflammation and autoimmune diseases can also cause elevation of serum CA19-9 [11]. Moreover, the study number in Takeshita's report was too small. Therefore the relationship between IEC and CA19-9 needs to be further clarified by strictly designed control study. Our study was designed to explore the diagnostic value of CA19-9 in IEC. Although we only recruited 42 patients in the study group, validity was guaranteed by excluding the confounding conditions mentioned above, and establishing a health control matched by gender and age. A cutoff value was also established with relatively high sensitivity and specificity.

Immunohistochemical study of EC with elevated CA19-9 disclosed positive staining mainly on the epithelial cells, subepithelial collagenous tissue and keratinous tissue. Therefore, Takeshita attributed the various levels of CA19-9 to the amount of the secretary glands, thickness of the capsule and location of the EC, and the existence of a blood brain barrier [6]. In line with this hypothesis, we found a positive correlation between CA19-9 level and tumor size. And unlike the commonly high expression levels of CA19-9 in extracranial disorders such as digestive system cancers, most IEC in our series were mildly or moderately elevated, with a median level of 20.3 ng/mL and IQR of 39.1, which might be attributed to theexistence of blood brain barrier. Except one case of ruptured IEC with intraventricular dissemination and severe aseptic meningitis, the barrier might be destroyed by inflammation and as a result the CA19-9 was extremely elevated. In the 9 subcutaneous ECs excluded, however, we obtained a mean CA19-9 level of only 6.03U/ml (data not displayed). Compared with IEC, subcutaneous EC does not have blood brain barrier, which suggests factors other than blood brain barrier may lead to this discrepancy. Anyhow, the abnormally elevated serum CA19-9 should arouse our attention of the possibility of IEC after a negative work-up for gastrointestinal system diseases.

In this study, we only analyzed the expression of CA19-9 in serum. For nervous system diseases, cerebrospinal fluid (CSF) usually has higher sensitivity and specificity than blood samples. In intracranial germ cell tumors, Allen found a positive rate of 34.5% in CSF compared with only 2% in blood [15]. In another study focusing on the value of CEA in differentiating intracranial metastasis from other neoplasms, Batabyal detected mean CSF CEA levels of 6.3 mg/ml, 0.92 ng/ ml, 0.31 ng/ml, 0.22 ng/ml in intracranial metastasis, intracranial primary tumor, benign tumor and normal control, while those of blood CEA levels were 5.2 ng/ml, 3.0 ng/ml, 2.7 ng/ml and 2.5 ng/ml respectively [16]. As an invasive technique, lumbar puncture is sometimes contraindicated in intracranial lesions causing mass effect, as it may lead to devastating brain herniation. In future study, after excluding the potential risks, CSF samples could be obtained to analyze the CA19-9 expression, which may further enhance the diagnostic value.

The limitations of this study are the relatively small study number and no strict follow up plan, which is due to the retrospective nature of this study. As a result, we did not set up validation cohort to verify the cutoff value, specificity and sensitivity. Postoperatively, DWI was not applied for evaluation, which might underestimate the residue rate and recurrence rate. Serum CA19-9 level was not dynamically checked after surgery, therefore unable to excavate the application of CA19-9 in prognostic evaluation and follow-up surveillance.

## MATERIALS AND METHODS

We recruited consecutive patients with IEC from the Neurosurgery Department, the Second Affiliated Hospital of Zhejiang University, Hangzhou, China, from 2009.12 to 2014.12. Approval for the study was obtained from the Ethics Committee of the hospital. The patient records were anonymized and de-identified prior to analysis. Patients with pathological confirmation of IEC were included. The exclusion criteria were: 1) they did not receive any preoperational radiological exam; 2) EC was located out of central nervous system; 3) no preoperative serum tumor biomarkers were obtained; 4) surgery was refused; 5) they were concomitant with other disease leading to abnormal tumor biomarkers such as lung cancer, gastrointestinal inflammation or tumor, genital system tumor. Healthy controls were selected with matched gender and age, ruling out disorders of gastrointestinal, biliary, genital and pulmonary system or other malignancies.

Data were abstracted from the medical charts. We recorded age, gender, past medical history, radiological studies including tumor size and location, level of preoperational tumor biomarkers, extent of lesion resection, pathological results and any recurrence during follow-up. Tumor size was estimated by the product of three dimensions [17]. Serum concentrations of tumor biomarkers, CA19-9, CA125, SCC and CEA, were measured by enzyme-linked immunosorbent assay (ELISA). Recurrence was defined as any reappearance of characteristic signal after gross total resection or enlargement of residue after subtotal resection from follow-up MRI.

Statistical analyses were done with SPSS for Windows (version 20.0) and diagrams were drawn with GraphPad Prism 5. Receiver operating characteristics (ROC) curves were constructed to assess the areas under the curves (AUCs) with 95% CI, with AUC between 0.5–0.7, 0.7–0.9 and > 0.9 indicating low, medium and high diagnostic value respectively. We investigated the optimum cutoff value by maximizing the Youden index. The sensitivity, specificity, positive predictive value (PPV), negative predictive value (NPV), positive likelihood ratio (PLR) and negative likelihood ratio (NLR) were calculated. For comparison between the two groups, the Mann-Whitney *U* test was used for continuous variables, while Kruskal-Wallis test for enumeration data. Logistic regression was used for multiple factor analysis. Linear regression was conducted to analyze the relationship between level of positive tumor biomarker and tumor size. We took *p* values lower than 0.05 (two sided) to be significant.

## CONCLUSIONS

To our knowledge, this is the first control study to report the clinically diagnostic relevance of serum CA19-9 in IEC. In this retrospective study we showed that IEC had significantly elevated serum CA19-9 level than the healthy controls. The cutoff value, specificity and sensitivity were also established by applying ROC curve. Our results indicate that serum CA19-9 could potentially be used to aid in the diagnosis of IEC. Further prospective study is needed to dig out the value of CA19-9 in postoperative evaluation and surveillance.

## Abbreviations

IEC: intracranial epidermoid cyst
CA19-9: carbohydrate antigen 19-9
CEA: carcinoembryonic antigen
CA125: carbohydrate antigen125
SCC: squamous cell carcinoma
ROC: receiver operating characterisitics
AUC: area under curve
HCG: human chorionic gonadotropin
AFP: α feto-protein
PPV: positive predictive value
NPV: negative predictive value
PLR: positive likelihood ratio
NLR: negative likelihood ratio
SD: standard deviation
CI: confidence interval
OR: odds ratio
CSF: cerebrospinal fluid
DWI: diffusion weighted image
